# Paradoxical worsening of *Emergomyces africanus* infection in an HIV-infected male on itraconazole and antiretroviral therapy

**DOI:** 10.1371/journal.pntd.0006173

**Published:** 2018-03-08

**Authors:** Kenneth Crombie, Zandile Spengane, Michael Locketz, Sipho Dlamini, Rannakoe Lehloenya, Sean Wasserman, Tsidiso G. Maphanga, Nelesh P. Govender, Chris Kenyon, Ilan S. Schwartz

**Affiliations:** 1 Department of Medicine, University of Cape Town, Cape Town, Western Cape, South Africa; 2 Department of Dermatology, University of Cape Town, Cape Town, Western Cape, South Africa; 3 Department of Anatomical Pathology, National Health Laboratory Service - Groote Schuur Hospital Branch, Cape Town, Western Cape, South Africa; 4 Division of Infectious Diseases & HIV Medicine, Department of Medicine, University of Cape Town, Cape Town, Western Cape, South Africa; 5 Clinical Infectious Diseases Research Initiative, Institute of Infectious Disease and Molecular Medicine, Department of Medicine, University of Cape Town, Cape Town, Western Cape, South Africa; 6 National Institute for Communicable Diseases-Centre for Healthcare-Associated Infections, Antimicrobial Resistance and Mycoses, National Health Laboratory Service, Sandringham, Gauteng, South Africa; 7 Sexually Transmitted Infectious Unit, Institute of Tropical Medicine, Antwerp, Belgium; 8 Max Rady College of Medicine, University of Manitoba, Winnipeg, Manitoba, Canada; 9 Global Health Institute, University of Antwerp, Antwerp, Belgium; Baylor College of Medicine, UNITED STATES

## Case presentation

**Fig 1:**
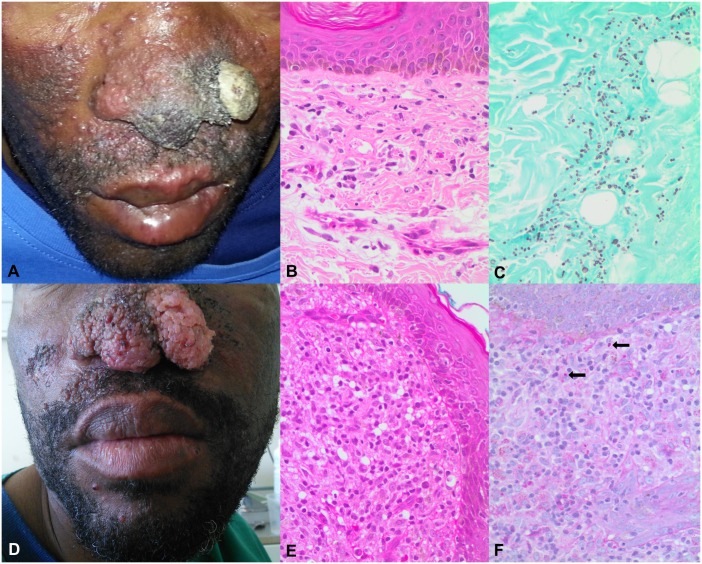
Clinicopathological features of a patient with HIV-associated emergomycosis initially and following antiretroviral-mediated immune reconstitution. (A) Clinical appearance of skin lesions at first biopsy. (B, C) High-power magnification histology of first skin punch biopsy showing (B) scanty macrophages and apoptotic nuclear debris around superficial dermal vessels (haematoxylin and eosin stain, x400) and (C) numerous small budding yeasts (Grocott methenamine silver stain, x400). (D) Clinical appearance at the time of second biopsy five months after the first. (E, F) High-power magnification histology of second skin punch biopsy showing (E) replacement of the entire dermis by dense sheets of foamy macrophages and admixed lymphocytes (haematoxylin and eosin stain, x400) and (F) isolated yeast-like structures (arrows; Periodic acid-Schiff stain, x400).

A 42-year-old male from an urban informal settlement in Cape Town, South Africa, was seen at a tertiary-care hospital for progressively enlarging lesions on his nose. He had initially presented to medical care 6 months earlier with a 3-month history of anorexia, weight loss, nonproductive cough, and nasal congestion. An HIV test was reactive, and his CD4 count was 32 cells/μL. He also had renal insufficiency with a creatinine clearance of 40 mL/min. Two weeks after diagnosis, he was initiated on antiretroviral therapy (ART) consisting of lamivudine (150 mg by mouth twice daily), stavudine (30 mg by mouth twice daily), and efavirenz (600 mg by mouth once daily). One week later, he developed widespread erythematous nodules and plaques on his face and a crusted mass on his nose (Fig 1A). A chest X-ray demonstrated a reticular opacity within the right middle lobe. A skin biopsy of a facial plaque demonstrated a minimal inflammatory response (Fig 1B). Methenamine silver staining revealed small (3–5-μm) yeast-like cells with occasional narrow-based budding (Fig 1C). Fungal culture of skin tissue and blood grew *Emergomyces africanus* (formerly *Emmonsia* sp.); identification was confirmed by sequencing of the internal transcribed spacer region (ITS). He was treated with intravenous amphotericin B deoxycholate (1 mg/kg) for 14 days. Thereafter, his ART was changed to lamivudine (150 mg by mouth twice daily), zidovudine (300 mg by mouth twice daily), and combination lopinovir/ritonavir (400/100 mg by mouth twice daily), and he was commenced on itraconazole (200 mg capsule orally once daily, to be taken with food) to continue for 1 year pending immune reconstitution.

Soon after initiating antifungal therapy, the patient’s constitutional and respiratory symptoms abated, and the cutaneous lesions improved over several weeks. However, the nasal lesions never completely resolved despite reported adherence to ART and itraconazole. Approximately 4 months after completing amphotericin B, his nasal lesions worsened over 1 month and he was referred to our tertiary-care centre for further evaluation.

On examination, multiple disfiguring verrucous lesions involving the entire bulb of the nose and smaller plaque-like lesions on his face were present (Fig 1D). His chest X-ray was normal. His full blood count and renal function were normal. His CD4 count was 110 cells/μL and his HIV-1 viral load was undetectable. A repeat skin biopsy was performed, and histopathological examination revealed the presence of an intense inflammatory reaction (Fig 1E). Periodic acid-Schiff staining revealed inconspicuous, scanty yeast-like structures (Fig 1F). From fungal culture of skin biopsy tissue grew a dimorphic fungus that was again identified phenotypically and confirmed by ITS sequencing as *E*. *africanus*.

Antifungal susceptibility testing was performed in parallel on the yeast phases of the 2 isolates cultured from skin tissue 5 months apart. The results were identical: the itraconazole minimum inhibitory concentration (MIC) was <0.008 μg/ml and 0.002 μg/ml using a broth microdilution method and E-test [1], respectively. Itraconazole therapeutic drug monitoring was not performed because it is unavailable in South Africa.

**Fig 2:**
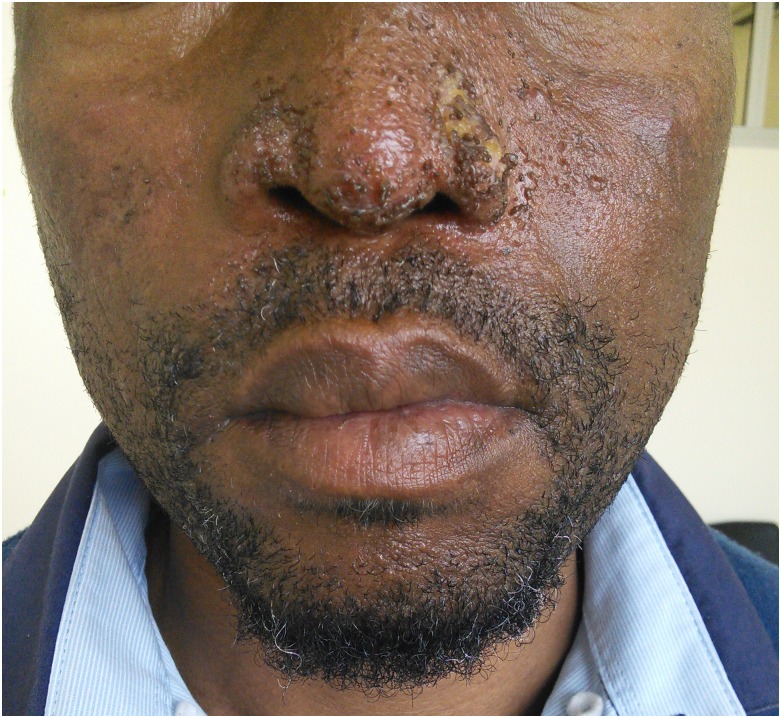
Clinical appearance of skin lesions after treatment with amphotericin B followed by itraconazole and concomitant prednisone tapered over three months.

The patient was treated again with intravenous amphotericin B deoxycholate for 14 days. Two days into therapy, prednisone 1 mg/kg daily was added with modest improvement in the facial lesions. On completion of amphotericin B, itraconazole (200 mg capsule by mouth once daily) was resumed, the prednisone was reduced to 30 mg by mouth daily, and the patient was discharged from hospital. At follow-up 2 weeks post-discharge, his facial lesions continued to improve (Fig 2). The prednisone was reduced to 15 mg daily for a further 2 weeks and then stopped.

After 10 months of HIV-1 virological remission and clinical remission of the skin lesions, the patient stopped his ART and itraconazole and relocated temporarily to another province for work. Three months later, he had returned to the Western Cape province and presented to care with fever, weight loss, and emesis. Blood testing demonstrated virological failure, with an HIV-1 viral load of 5.3 log_10_ copies/mL. Five days later, he was hospitalized for severe sepsis; he was pyrexic and had severe anemia with a hemoglobin of 4.5 g/L. Sputum cultures, real-time PCR (GeneXpert MTB/Rif, Cepheid Inc., Sunnyvale, CA), and urinary lipoarabinomannan (Determine TB-LAM Ag, Alere, Waltham, MA) were all negative for *Mycobacterium tuberculosis*. Fungal and mycobacterial blood cultures, cerebrospinal fluid examination by Gram stain, culture, real-time PCR for *M*. *tuberculosis*, and cryptococcal antigen (IMMY CrAg LFA, ImmunoMycologics, Norman, OK) were all negative. He received a blood transfusion and was treated empirically with broad-spectrum antibacterials and amphotericin B but deteriorated further, and on day 6 of hospitalization, he died. A post-mortem examination was not performed, and the cause of death remains uncertain.

## Discussion

South Africa has the highest burden of HIV worldwide, with approximately 7 million people living with HIV; however, only half of those eligible for ART are accessing treatment [2,3]. In addition, late-stage disease at presentation is common, with more than a third of patients presenting with CD4+ counts less than 100 cells/mm^3^ [4]. *E*. *africanus* is a recently-described species of dimorphic fungus that causes an AIDS-related systemic mycosis known as emergomycosis (previously disseminated emmonsiosis) in South Africa [5–7]. Disease most commonly involves the skin but can also affect the lungs, liver, and bone marrow [7]. Skin lesions have been reported to erupt or become more numerous after ART initiation, suggesting that an unmasking immune reconstitution inflammatory syndrome (IRIS) is frequently involved [7,8].

This report illustrates a case of emergomycosis complicated by progression of lesions and persistence of culturable fungus after amphotericin B and nearly 6 months of itraconazole and despite ART-mediated immune reconstitution. The histopathological observation of few yeasts and abundant inflammatory response in the patient’s second skin biopsy (compared to the findings of abundant yeasts but little inflammation noted on the biopsy at initial presentation), combined with the observed clinical improvement with a regimen that included prednisone, suggest that a paradoxical IRIS (apparent clinical worsening of disease previously controlled upon immune recovery, e.g., ART-mediated CD4+ T-cell lymphocyte repletion) may have contributed to this presentation. Paradoxical IRIS has been reported for other systemic mycoses including cryptococcosis [9] and occasionally other endemic mycoses like histoplasmosis [10,11] and coccidioidomycosis [12].

On the other hand, treatment failure is suggested by the isolation of *E*. *africanus* from the second biopsy of affected skin despite extensive prior antifungal therapy. Parallel in vitro antifungal susceptibility testing excluded itraconazole resistance, implying that the clinical deterioration may have been due to inadequate itraconazole exposure. The possibility of poor adherence to medication cannot be excluded.

Itraconazole is commonly recommended for long-term management of endemic mycoses in immunocompromised patients [13,14], and it has recently been added to the World Health Organization (WHO’s) Essential Medicines List [15]. However, clinicians should be aware of variables that can affect plasma concentrations of this drug [16]. Firstly, bioavailability of the capsule formulation is poor and can be affected by a number of conditions [17]. Bioavailability is enhanced by gastric acidity [18] and reduced 40% in the fasting state compared to when taken with food [16]. Absorption is reduced by 50% in patients with AIDS compared to healthy volunteers [19], possibly mediated by AIDS-related hypochlorhydria [20]. Secondly, itraconazole is a substrate of cytochrome P450 3A4 isoenzyme; consequently, drug–drug interactions are a concern [21]. Protease inhibitors (PIs) such as ritonavir cause increased serum concentration of itraconazole, and elevated levels have been linked to toxicity, notably prolongation of the QT interval [22]. Non-nucleoside reverse transcriptase inhibitors (NNRTIs) cause decreased serum concentrations of itraconazole [21]. Inadequate levels have been associated with poor treatment outcomes for some mycoses [20,23], and thus coadministration is not recommended [24]. Integrase inhibitors, however, are not expected to interact with the pharmacokinetics of itraconazole and may therefore be useful in this setting [25,26]. Given the difficulty in predicting plasma itraconazole levels from dosing, therapeutic drug monitoring is strongly recommended for itraconazole by the British Society for Medical Mycology [27]. Without access to therapeutic drug monitoring in sub-Saharan Africa and other resource-limited regions, and in the absence of robust population-pharmacokinetic data in Africans with advanced HIV infection, clinicians must consider all factors that may affect plasma concentrations and response to therapy, including known interactions with PIs and NNRTIs [26]. Patient education on the importance of adherence to prolonged therapy despite symptomatic improvement, emphasis on strategies—such as coingestion with food—to enhance bioavailability of itraconazole tablets, and close follow-up are essential to optimize the likelihood of successful clinical outcomes.

## Ethics statement

This study was approved by the University of Cape Town Faculty of Health Sciences Human Research Ethics Committee (HREC 138/2014). Consent to publish this case report, including photographs, was provided by the patient’s wife.

**Key learning points**

- *E*. *africanus* causes a disseminated endemic mycosis among immunocompromised hosts in southern Africa.
- Systemic fungal infections should be considered in patients with advanced HIV disease who present with widespread skin lesions that are accompanied by systemic symptoms and/or abnormal chest radiographs.
- Itraconazole exposure can be increased in patients taking PIs and decreased in patients taking NNRTIs and rifampin (among other agents).
- The lack of itraconazole therapeutic drug monitoring in South Africa may result in patients being improperly dosed.
- The clinical manifestation of cutaneous lesions caused by *E*. *africanus* can depend on the patient’s immune status and can thus be dynamic.
